# Reaching “covidianidad”: A qualitative study of the impact of the COVID-19 pandemic on the perceived mental health of health care workers in the Dominican Republic

**DOI:** 10.1371/journal.pgph.0002652

**Published:** 2023-12-01

**Authors:** Pamela Baez Caraballo, Simone Schriger, Jessica Escober, Ana Acevedo, Antonio García Alejandro, Mina Halpern, Elizabeth Lowenthal

**Affiliations:** 1 Clínica de Familia La Romana, La Romana, Dominican Republic; 2 Department of Psychology, University of Pennsylvania, Philadelphia, PA, United States of America; 3 School of Public Health, University of Michigan, Ann Arbor, MI, United States of America; 4 Department of Pediatrics, Global Health Center, The Children´s Hospital of Philadelphia, Philadelphia, PA, United States of America; 5 Department of Pediatrics, University of Pennsylvania Perelman School of Medicine, Philadelphia, PA, United States of America; 6 Department of Biostatistics, Epidemiology, and Informatics; University of Pennsylvania Perelman School of Medicine, Philadelphia, PA, United States of America; The University of British Columbia, CANADA

## Abstract

We aimed to explore how the COVID-19 pandemic affected the lives of healthcare workers (HCWs) in the Dominican Republic. We also aimed to identify the types of resources that HCWs felt were needed to support their mental health. We used purposive and convenience sampling in four health centers in the eastern Dominican Republic to recruit 28 HCWs (doctors, nurses, psychologists, and community health workers) between April 2021 and August 2021. Through semi-structured interviews, we elicited HCWs experiences during the pandemic and how they felt these experiences impacted their mental health. Interview transcripts were analyzed using an inductive/deductive thematic approach. Main stressors experienced during the pandemic by HCWs and their sequelae included anxiety due to misinformation and uncertainty, fear of the disease, the robustness of pandemic-related changes they faced in their work and daily life, and COVID-19’s economic impact. HCWs reflected on protective factors that transformed their acute sense of crisis felt at the beginning of the pandemic into what HCWs referred to as “covidianidad [everyday COVID]”, a situation that became manageable through mechanisms including social support, professional motivation, positive work environment and resilience. Lastly, HCWs identified stigmatization of and limited access to mental health services as challenges to supporting their mental health. While Dominican HCWs were vulnerable to the challenges posed by COVID-19 in sustaining their mental health, for many, the situation became manageable through the evolution of “covidianidad.” Further research and interventions are needed to reduce stigmatization of mental health services and foment a positive environment for HCWs’ mental health, to promote resiliency to future challenges.

## Background

The coronavirus disease 2019 (SARS-CoV-2, COVID-19) exposed how healthcare systems were ill-equipped to respond to the rapid spread of an infectious pandemic [1, 2], despite countries’ efforts to establish nationwide safety measures [2, 3]. The general population experienced psychological burdens such as fear of the disease and losing loved ones, economic instability, and living within the constraints of preventative measures [4–6]. Healthcare workers (HCWs) were especially vulnerable [7], since they were the first responders to a highly contagious, unfamiliar, and potentially lethal disease [8, 9].

COVID-19’s high infectivity rate caused HCWs throughout the world to experience overwork, distress, anxiety, and feelings of powerlessness during the initial phase of the pandemic [8, 10–12]. Factors associated with higher psychological burden on HCWs include fear of contracting or spreading COVID-19 to close family members, personnel shortages, insufficient personal protective equipment (PPE), stigma, misinformation and uncertainty about the future [5, 9, 13–18]. Protective factors for HCWs’ mental health in many settings include clear understanding of safety guidelines, professional motivation, religion, sufficient PPE, strong health center leadership and social support [9, 18–21].

The Dominican Republic (DR) is a Spanish-speaking, middle-income country located on Hispaniola, an island shared with Haiti in the Caribbean [22, 23]. It has approximately 11 million habitants [24, 25],with an estimated 1.5 doctors per 1,000 habitants [24], less than recommended by WHO to sustain population health [26]. In response to the pandemic, the DR implemented three months of quarantine from March to June 2020, during which the general population was instructed to remain at home for 16 hours of the day, followed by 13 months of 12-hour lockdown. Additional safety measures included limiting use of mass transportation services and social activities (e.g., churches gatherings at 50% capacity), and creating a telephone helpline to provide access to mental health services [27]. Of 6,800 individuals who utilized this helpline, 27% were HCWs [28]. Overall, the main reasons for calls to the helpline were anxiety (32%), stress (20%), and depression (9%). Reports in the DR found stress as the most common burden among HCWs [11, 29], while another report identified fear of COVID and contact with COVID-19 patients as predictors of acute stress in the Dominican HCW population [30]. However, data about the pandemic’s impact on Dominican HCW’s perceived mental health status remains limited. Thus, we aimed to explore HCWs’ experiences during the COVID-19 pandemic, and its effect on HCWs’ perceived mental health. To our knowledge, this is the first qualitative study in the DR exploring HCWs’ perceived mental health during the COVID-19 pandemic.

## Methodology

### Ethics statement

This study was approved by the Consejo Nacional de Bioética, in the Dominican Republic and the Institutional Review Board of the Children’s Hospital of Philadelphia in the United States. Informed written consent was obtained from participants prior to data collection.

### Study setting and recruitment

Our qualitative study was conducted in La Romana and San Pedro de Macorís, two provinces in the eastern DR, between April 2021 and August 2021. HCWs were interviewed from four different centers: two outpatient centers (Clínica de Familia La Romana and Centro de Salud Divina Providencia, two not-for-profit organizations providing primary care services specialized in vulnerable populations including migrants, people living with HIV and children) and two inpatient centers (Hospital General El Buen Samaritano, a non-for profit health center and the provincial public Hospital Provincial Arístides Fiallo Cabral, chosen for being the only two non-privatehospitals in La Romana with a COVID-19 unit available). We used criterion purposive sampling for the inpatient centers, specifically inviting all HCWs working in COVID-19 units and ICU units to obtain the most information-rich data from participants [31–34]. For the outpatient centers, where all staff had similar involvement with COVID-19 patients, we participated in the centers’ staff meetings to invite any available staff who met inclusion criteria to participate in the study. Inclusion criteria were being a Spanish-speaking doctor, nurse, community health worker (CHW) or psychologist over the age of 18 in any participating health center, practicing as a HCW since at least March 2020, and being willing to participate.

### Data collection

A semi-structured interview guide (S1 Text) was pre-evaluated by a small group of HCWs to ensure clarity and appropriateness. The guide used open-ended questions such as: ‘Please describe how your work has changed since the beginning of the COVID-19 pandemic’ and ‘What would you say is your biggest motivation to continue working as a HCW since the beginning of the COVID-19 pandemic?’ These questions were followed by probing and exploratory inquiries about how HCWs’ thoughts, feelings, concerns and coping mechanisms had changed since the start of the pandemic compared to the time of the interviews. Researchers trained in interviewing techniques (PB and CG) conducted all semi-structured interviews face-to-face in a private room at each health center, taking safety and social distancing measures into consideration. CG was not previously known to any of the interviewees, while PB was known to interviewees at Clínica de Familia La Romana but not at the other study sites. Interviews were audio recorded and ranged from 30 to 60 minutes.

We anticipated enrolling approximately 24–32 participants (6 to 8 participants per site) [35]. To establish sample size, we followed Fossey’s suggestion, seeking enough data to fully describe a phenomenon and revising the data obtained until trends occur, and we feel gathering more data would yield similar results [36, 37]. We followed Guest et al. methodology for calling data saturation [38], where interviewers met every 3–5 interviews, and new topics were discussed and added to pre-existing categories found. Guest suggests that little variation in the data is found after the first 12 interviews. In our case, new information was limited at interview 18 and no more new information was found when reviewing about 23 interviews. To ensure data saturation [39], we conducted five more interviews, resulting in a total of 28 interviews. The final five interviews similarly did not yield new themes. The interviews were de-identified and transcribed verbatim in Spanish by a third party employed by the research team. To further maintain confidentiality, demographic data for the interviewees will be presented collectively. Key quotes extracted from the transcripts were translated verbatim by PB, then back translated using Deepl [40], a web-based translation service, and confirmed by PB and MH, who are both bilingual.

### Data analysis

We used thematic analysis (TA) to elucidate our findings, following a deductive-inductive approach in accordance with Braun and Clarke’s six steps to thematic analysis consisting of data familiarization and generating codes, which then are merged into themes and iteratively reviewed for outlining, defining, and identifying excerpts to illustrate each theme [41, 42]. We chose thematic analysis because this methodology allows for the exploration of common themes across cases, accommodates for a large sample size, and allows for interpreting thematic statements into calls for action [43]. Data analysis was done by three Spanish-speaking coders (PB, AA and JE) simultaneously coding transcripts independently in an iterative fashion. Analysis began by reading the transcriptions several times to immerse the coders in the data, prior to sharing their main findings and initial impressions. An initial codebook with 76 codes emerged from the initial review of the transcripts. During periodic meetings between all coders and EL, the codebook was iteratively revised and discussed to obtain meaning and context from the experiences given by the interviewees [44]. While the coders first coded line by line, they simultaneously noted links between themes. Coders referred to the codebook text when contradictions arose but also sought to be open to new and more nuanced meanings as they arose in the data. Themes were refined throughout to reflect the data and two of the interviewees were contacted to double-check meaning and local context of the data provided. We then did final review of the transcripts, to further confirm meaning and interpretation of our data [44, 45]. We used NVivo v.12 for data analysis.

Analytic rigor was ensured by several steps. Credibility was obtained in the following ways: 1) through data immersion by the coders, overlapping 8 (22%) of the interviews between the coders for double/coding and inter-coding comparison [46, 47]; 2) by adopting an established research methodology (TA); 3) by debriefing sessions between coders and EL, an experienced qualitative researcher mentoring the process of the codebook creation and refinement of data interpretation based on new findings; and 4) through member checking. Transferability can never be fully assured, but including different settings and occupations gave the opportunity to understand experiences in a way that can be a reflection of the target population in the Eastern DR [48]. The research team kept a record of all notes and changes to ensure objectivity and replicability of the coding process. Key quotes extracted from the transcripts presented in this article were translated by PB, then back translated using Deepl [40], a web-based translation service, and confirmed by PB and MH, who are both bilingual.

## Results

We interviewed 28 HCWs, with half of the interviews in outpatient settings and half in inpatient settings (Table 1). Most participants identified as female (25, 89%). Almost half (13, 46%) were doctors, 7 (25%) were nurses, 5 (18%) were community health workers and 3 (11%) were psychologists. The median age of participants was 33 (IQR 25–43) years.

**Table 1:** Demographic characteristics of participants.

	Outpatient center	Inpatient center	Male	Female	Median age (IQR)
Doctors	3	10	2	11	34 (28–41)
Nurses	3	4	0	8	30 (26–43)
Psychologist	3	0	1	2	40 (36–49)
Community health workers	5	0	0	5	36 (30–46)
Total	14	14	3	25	33 (28–43)

Inter-coder reliability was obtained from eight (22%) overlapping interviews (*k* = 0.86, 94% agreement) amongst three coders. Based on the structure of the interview guide, results were categorized into four main themes. Subthemes emerged from the data itself: stressors (5 subthemes), protective factors (4 subthemes), adaptation process (5 subthemes), and future perspectives to sustain HCWs’ mental health (2 subthemes; see Table 2 for thematic framework).

**Table 2:** Thematic framework for the COVID-19 impact on Dominican health care workers’ perceived mental health.

Theme	Subtheme	Common topics relating to subtheme	Quotes
Stressors	Lack of knowledge caused anxiety among HCWs	Misinformation and uncertainty	“At the beginning, we did know how it spread, what could cause it, how to manage it, if people would die… obviously, we were scared.” -Female doctor 1, inpatient site 2
		Pandemic was new for everyone.	“The pandemic was something new, and unfortunately, nobody was ready for it.” -Female doctor 2, outpatient site 1
		Sense of disbelief at the beginning of the pandemic	“People just started dying, many per week, because of COVID. That’s when I thought ‘this is for real’.” -Female doctor 1, inpatient site 1
	Fear of disease	Fear of relatives becoming infected	“My biggest fear was for my parent to be infected.” -Female nurse 4, inpatient site 2
		I or my relative have comorbidities	“My husband is diabetic, and my son had a kidney transplant. So, my fear was them being infected.” -Female nurse 1, outpatient site 1
		Fear of me taking COVID to my relatives	“My biggest fear was my family getting COVID, and knowing I took it to them.” -Female doctor, inpatient site 2
		Stigma toward HCWs	“At the beginning, neighbors wouldn’t even come close.” -Female doctor 1, inpatient site 1
	Changes in their personal life	Social distancing and isolation	“The only stress I felt was when I couldn’t leave my house.” -Female nurse 2, outpatient site 1
		Incorporating safety measures into daily life	“When you get home, it is taking off clothes before coming in.” -Male doctor 1, inpatient site 1
	Changes in their professions	Feeling burnout	“Lots of work. That’s totally my source of stress, so much work.” -Female nurse 2, inpatient site 2
		Using PPE and keeping safety measures around patients	“You need to cover head to toe to see patients now.” -Female doctor 2, inpatient site 2
		Death due to COVID	“Seeing people die, even young people, it hurt.” -Female nurse 3, inpatient site 2
		Patients during the COVID-19 pandemic	“Patients we’re getting are very affected, so it’s a double effort. You need to handle the effects of the pandemic and their daily lives.” -Female psychologist, outpatient site 2
	Economical factors	Loss of income in the household	“I was affected, because I lost my second job at the beginning of COVID.” -Female doctor 1, inpatient site 2
		Low Dominican HCW salaries	“People think that since you´re a doctor, you´re rich, but we’re struggling.” -Female doctor 2, outpatient site 2
Protective factors	Motivation to continue as HCW	Patients’ health	“You feel this satisfaction to see them healthy, and they thank you. It’s very gratifying.” -Female doctor 3, inpatient site 2
		I always wanted to be a HCW	“I always wanted to be a doctor; I’ll always carry that with me.” -Female doctor 2, outpatient site 2
	Social support	Family	“If anything, I can count on my family.” -Female nurse 4, inpatient site 2
	Positive work environment	Teamwork	“The environment, it helps so much when there’s more positive than negative in your team.” -Female doctor 1, outpatient site 2
		Open communication	“Leadership needs to explain changes. When you don’t have an explanation, it’s frustrating.” -Female CHW 4, outpatient site 1
		Positive response to an epidemic	“We got so much support, information about the disease, prevention since the pandemic began.” Female CHW 2, outpatient site 1
	Available vaccine	An emerging hope	“I got two shots already, and I’m so happy there’s this light at the end of the tunnel.” Female psychologist 2, outpatient site 1
Adaptation process	Adaptating through learning	More knowledge, less stress	“We know much more than before, so it’s easier to relax.” Female doctor 1, outpatient site 2
		Identifying sources of information	“We had to be very careful about the information we read, what to believe in.” Female doctor 2, inpatient site 1
	Digital tools	Using digital tools for healthcare	“I keep in touch, but you know… digitally, not in person.” Female doctor 2, outpatient site 2
		Using telemedicine	“Now instead of being at work, most of it is over the phone.” Female CHW 2, outpatient site 2
	Implementing support measures	More time with their families	“Spending time with my family at home made it easier to cope with stressful situations.” Female CHW 1, outpatient site 1
		Greater chance to self-care	“I take time for myself, sit in a quiet place, breathe, music.” -Female psychologist 1, outpatient site 1
	Increasing vaccine availability	A ray of hope	“We can hope this will be over soon, especially with the vaccine.” -Female CHW 2, outpatient site 1
		Impact of getting vaccinated	“Being protected with the vaccine, it helps a lot.” -Female doctor 4, inpatient site 1
	Covidianidad: learning to live with COVID	Feeling that this is the “new normal”	“We’re getting used to it, to live with COVID.” -Female nurse 4, inpatient site 2
Future perspectives	Challenges	Population behavior	“Making people understand and cooperate is a challenge.” -Female doctor 4, inpatient site 1
		Vaccine for everyone	“First of all, we all need to vaccinate. Everyone!” -Female nurse 1, outpatient site 2
	Dominican population’s mental health	Stigma on mental health services	“We all need help, but that doesn’t mean we’re crazy.” -Female doctor 2, inpatient site 2
		Access to mental health services	“We don’t have a dedicated program for HCW to speak with mental health providers if we needed it.” -Female nurse 3, inpatient site 2

### Theme 1: Stressors

#### Subtheme A: Lack of knowledge and uncertainty caused anxiety among HCWs

Anxiety was most prominently expressed in relation to lack of knowledge, uncertainty about the future and sense of disbelief during the early stages of the pandemic. Much of the initial anxiety around COVID-19 was related to information overload from informal sources (media, websites, relatives, etc.) combined with lack of readily available reliable information about COVID-19 management from official sources, which made managing COVID-19 patients increasingly difficult for HCWs. As such, in the initial stages of the pandemic, all HCWs included in our study felt overwhelmed by how fast the disease spread, the increasing length of the pandemic, and an overall sense of helplessness and disbelief at facing a global pandemic.

*“There was a lot of anxiety at the beginning, a lot of information, a lot of misinformation. What was valid today was not valid tomorrow… I don’t think anybody was ready for that moment when the pandemic began.” -Female psychologist 2, outpatient site 1*

*“We were working against something we knew nothing of, ‘God, when I get a patient, how do I manage them?’” -Female doctor 3, inpatient site 1*

*“It hit me really hard, I never thought COVID would happen here, I’ve never seen anything like this, only like, in the movies.” -Female CHW 4, outpatient site 1*

#### Subtheme B: Fear of the disease as a constant stressor

The most recurrent topic that was raised related to fear of disease was fear of relatives becoming infected, regardless of HCWs’ clinical setting and occupation. Nonetheless, inpatient interviewees described greater fear of ‘carrying the disease home’ to their loved ones than outpatient interviewees. They were especially concerned about the elderly given their vulnerability for COVID. A direct consequence of this fear was HCWs adopting extensive precautions to avoid infection. HCWs described becoming more fearful and anxious due to their inability to keep the pandemic from their minds.

*“My mother has high blood pressure and diabetes. I work in the COVID unit. I feel this terrible fear of bringing COVID home.” -Female nurse 3, inpatient site 2*

*“I have relatives with health conditions that the virus hits harder; my greatest fear is them dying.” -Female doctor 2, inpatient site 1*

*“My biggest fear was carrying the disease to my family.” -Female nurse 1, inpatient site 1.*

*“My hands were dry, I washed with alcohol so much, to avoid getting COVID.” -Female doctor 2, outpatient site 1*

An unexpected finding surrounding fear of the disease was HCWs’ fear of having their children become infected, despite understanding severe cases of COVID-19 in children were rare. This fear was often related to the possibility of a child presenting as asymptomatic and passing the infection to someone else.

*“Children are a source of contagion. Children don’t know how to take care of themselves.” -Female nurse 4, inpatient site 2.*

*“It’s not as hard on children, but I’m scared my daughter gets COVID-19 and passes it to my grandma.” Female nurse 2, inpatient site 2*

Another aspect of fear of COVID-19 relates to HCWs experiencing stigma from the general population. About half of the interviewees expressed either witnessing or experiencing some form of stigma related to their profession, typically stemming from the population perceiving HCWs as ‘carriers of COVID’, whether the HCWs worked in COVID-19 wards or not. HCWs’ reflections, however, were sympathetic and understanding of these attitudes.

*“People said things like ‘I don’t want her here. She comes from the COVID hospital and can infect us’ even when you don’t work in the COVID area. I understand they’re trying to protect themselves.” -Female nurse 2, outpatient site 1*

*“They label you like you have COVID-19, and no one will even say ‘hi’ to you.” -Female CHW 2, outpatient site 2*

*“People walk away from you in the streets, even. But I understand that they’re taking care of themselves.” -Female doctor 2, outpatient site 2*

#### Subtheme C: Changes in their personal life due to safety measures

HCWs highlighted important changes in their personal lives that resulted from incorporating safety measures into their lives. Most HCW expressed struggling with social distancing and isolation during the pandemic, feeling duress by either having their social lives reduced, or being away from their family. These feelings were particularly present among inpatient HCWs, where working in the COVID-19 specialized unit often included being physically away from their families, further increasing their sense of isolation.

*“I stopped spending time with my daughter and my parents. You could have COVID even without symptoms, so I need to act like I always have it.” -Male doctor 1, inpatient site 1*

*“Not being able to go out, visit people…. It made me angry.” -Female CHW 1, outpatient site 1*

*“The distancing makes us feel locked up. We [Dominicans] are used to hanging out, going out, greeting warmly with hugs and kisses.” -Female doctor 4, inpatient site 1*

*“Couldn’t go out, spend time with our families and friends, no way to release stress, everything was just COVID, COVID, COVID.” -Female CHW 2, outpatient site 1*

Additional stressors include how these changes affected daily activities such as buying groceries, paying bills, not being able to do leisure activities anymore and taking on more responsibilities at home to care for their families, further cementing life in the pandemic outside of their work times.

*“I never used to do grocery shopping, I hate that, but I was the only one who could, to protect my parents.” -Female psychologist 2, outpatient site 1*

*“You stop doing things like going to the gym or getting your hair done because you don’t know if they’re vaccinated, or exposed.” -Female doctor 4, inpatient site 2*

#### Subtheme D: Profession/changes in work environment as HCWs

The main source of workplace stress was the higher workload for HCWs. This increase in workload was more acute in the inpatient setting, where most HCWs reported feeling pressured and burned out by a combination of greater number of patients, new responsibilities in their workplace, and managing COVID-19 patients. Patient management and death were often described as one of the hardest things to witness by doctors and nurses, causing feelings of sadness in their workplace and making them question their place as HCWs. For some inpatient doctors, burnout manifested as somatization of symptoms, or frustration associated with feeling like their efforts were not enough to face the COVID-19 pandemic, since COVID-19 patients were perceived as frail and delicate, in need of constant vigilance and monitoring as their health could deteriorate at any moment.

*“Working in the COVID unit, I felt burnout. It got full, up to 12 patients for a nurse, many of them critical.” -Female nurse 4, inpatient site 2*

*“Because of the stress, all the work, the fatigue, sometimes we even feel headaches, body aches.” -Female doctor 1, inpatient site 1*

*“It doesn’t matter how much you monitor vitals, oxygen, blood pressure, kidneys… they deteriorate, don’t respond to the treatment. Seeing patients die, it wears you down. It makes you more likely to be depressed.” -Female doctor 4, inpatient site 2*

*“It’s very painful. You do all you can, but if they die, you feel powerless.” -Female nurse 2, inpatient site 2*

Another theme regarding changes and stressors in their work environment included using PPE. All HCWs expressed discomfort and difficulty with using PPE while taking care of patients. Most of these challenges related to how PPE and safety measures limited HCWs’ ability to perform a complete evaluation on the patients; doctors and nurses specifically shared how PPE and safety measures led to treating patients in a manner that restricted some aspects of their effectiveness as providers, such as interfering with creating a strong patient-provider relationship.

*“Examining the patients is very different now because you need to examine them with a mask on, gown, gloves. We still do physical examinations, but the patient-provider connection is not the same.” -Female doctor 4, inpatient site 1*

*“That closeness isn´t there anymore. We need to take care of the patient and ourselves; we don’t know who’s infected or not.” -Female doctor 2, inpatient site 1*

*“Wearing the masks is the worst. You can’t breathe with it on.” -Female CHW 2, outpatient site 1*

*“Wearing all that was like a punishment. If a patient took a turn and you weren’t wearing it, you lost time.” -Female doctor 2, inpatient site 2*

A topic that arose unexpectedly from interviewees revolved around the patients’ and patients’ relatives’ behavior. Doctors and nurses specifically highlighted difficulty around sharing information about COVID-19 with patients and their family members, explaining that patients and their relatives often expressed disbelief and became upset when informed of a positive COVID-19 test, causing HCWs to become apprehensive of patients’ and their relatives’ reactions while delivering COVID-19 related news, as this reaction further hindered their performance as HCWs. Similar difficulties included discussing the new visiting regulations due to COVID-19 safety measures with patients and their relatives, as relatives insisted on staying close to the patients during their hospital stay, despite the risk of being infected. Such attitudes from patients and relatives added a new level of complexity which the HCWs had to adapt to quickly.

*“A lot of patients even leave because they refuse to believe they have it [COVID].” -Female doctor 3, inpatient site 1*

*“Relatives don’t believe you and you have to be careful how you explain things to them; they don’t understand their relative has COVID and they need to protect themselves.” -Female doctor 3, inpatient site 2*

*“When we tell the relatives ‘You have to go home; you can’t be here,’ they get upset and say things like ‘you have no sympathy; you’ve never had relatives with COVID.’” -Female doctor 4, inpatient site 2*

At the time of the interviews, HCWs expressed that many of these reactions were still present, albeit less emphatically than during the early months of the COVID-19 pandemic. Overall, HCWs’ perception of their patients was described as “tense, nervous and scared.” HCWs shared that even subconsciously, this behavior influences HCWs’ demeanor and way of approaching patients, creating a sense of stress overload in their healthcare setting for everyone.

*“It’s like a stress chain. The population we’re treating is already stressed. So, you need to try harder to keep things stable and working, and that makes it more stressful” -Female psychologist 2, outpatient site 1*

*“Patients immediately think they’ll die. They see other sick people dying and it scares them.” -Female nurse 2, inpatient site 2*

#### Subtheme E: COVID-19’s effect on the economy as an additional burden

HCWs were concerned about their finances due to the consequences of COVID-19. Concerns expressed included fear of unemployment and reduced salaries, for both HCWs and their family members, with many HCWs becoming the only source of income in their household. This stressor was mainly emphasized by outpatient HCWs among our interviewees. Outpatient HCWs were subject to reduced salaries, loss of employment and, thus, financial insecurity during the early stages the COVID-19 pandemic, even while handling greater numbers of patients and experiencing burnout. However, when discussing economic burdens, both inpatient and outpatient HCWs expressed feeling that they receive insufficient compensation for their work.

*“My biggest stress is that my husband is unemployed now.” -Female CHW 1, outpatient site 2*

*“Many relatives are not working anymore, or get sick because of COVID. So, you have more pressure to get money for food, medication, and so on.” -Female psychologist 1, outpatient site 1*

*“Expenses are the same, but the income is less, and that makes it hard…. It takes longer and its harder to meet responsibilities.” -Female doctor 2, inpatient site 2*

*“We only received a portion of our salaries for a long time.” -Female nurse 2, outpatient site 1*

### Theme 2: Protective factors for HCWs’ mental health

#### Subtheme A: Motivators to continue duties as an HCW

HCWs were asked to identify their main motivation to continue working during the COVID-19 pandemic. They expressed a combination of factors that included patients’ health and a sense of calling and duty as HCW. While both factors were conceptually linked together by many HCWs, patients’ health as main motivator was more commonly singled out among outpatient HCW, while inpatient HCWs perceived their motivation as a sense of calling and duty as HCWs, described as having chosen to care for patients’ health and feeling great satisfaction witnessing patients’ recovery and return to their families.

*“For anyone working in healthcare, I think the motivation is helping patients everyday.” -Male psychologist 1, outpatient site 2*

*“My biggest motivator is seeing a patient who had coughing and breathing difficulties, visibly sick, get back on their feet and go back to their home and families.” -Male doctor 1, inpatient site 1*

*“We do this by vocation, we’re not afraid to help people when they’re sick.” -Female nurse 2, inpatient site 1*

*“I think [healthcare] is something you do from the heart; if we don’t feel it, we can’t do it.” -Female CHW 2, outpatient site 2*

None of the HCWs described feeling obligated to perform as an HCW. Instead, they described continuing to work as fulfilling their commitment as a HCW despite the stressors, and how COVID was a situation that became manageable through social support, professional motivation, positive work environment and resilience.

*“I knew we had to face the pandemic because it is what we prepared for as healthcare professionals.” -Female nurse 1, outpatient site 1*

*“We chose to work in the healthcare area. We can’t let ourselves feel defeated. We have to set an example.” -Female doctor 2, outpatient site 2*

#### Subtheme B: Receiving social support

HCWs mentioned using several activities and coping mechanisms during the initial stage of the pandemic to manage their mental health, including watching movies, going to church, and listening to music. However, when asked about protective factors, the most common answer was spending more time with their families and receiving emotional support from loved ones to continue their work as HCWs.

*“I can count on my mom, my dad even if he’s away, my husband, aunts and uncles… my family has remained together.” -Female doctor 1, outpatient site 2*

*“My mother, my partner, and children- they give me a lot of love and support. They say, ‘take care of yourself; we know you’re there [at work].” -Female doctor 1, inpatient site 1*

Expanding on unexpected results about protective factors, it was notable that only a minority of our HCWs mentioned religious or faith-related activities as protective factors or coping mechanisms to protect their perceived mental health.

#### Subtheme C: Positive work environment

HCWs also reflected on the importance of work climate. Despite enduring COVID-related stress and anxiety, interviewees perceived a sense of companionship and support from their colleagues, doing things such as sharing new information with each other, covering for an absent colleague, or assisting each other in their work. Such actions allowed HCWs to feel as though the burden was shared by all colleagues and, thus, easier to carry.

*“We work as a team, no matter what. If I notice my nurse doing something [they shouldn’t], or I’m missing a lab result, we’re always communicating.” -Female doctor 2, outpatient site 2*

*“All of us work to keep the unit going. Not everything falls on the doctor; all of us, nurses, cleaning staff, security, we all know what to do.” -Female doctor 4, inpatient site 2*

On that same sentiment, HCWs stressed the importance of appropriate response and leadership during times of crisis, which allowed them to better perform their duties as HCWs in their work area. Highlighted aspects of a positive organizational response included establishing safety protocols, providing sufficient PPE, educational activities, and open communication among coworkers and with leadership. It is notable, however, that positive feedback like “organizational response made me feel safer” came more from HCWs in outpatient than in inpatient settings.

*“It’s very important that all leaders get involved with [creating] procedures, figuring out what can improve, what they can change for us to be able to do our work.” -Female psychologist 2, outpatient site 1*

*“We are already pressured by patients, relatives; it’s gratifying when your team tries to understand us, the risks we take, and makes things easier for us.” -Female doctor 1, outpatient site 2*

*“Every time there is a meeting, we always get talks about safety protocols, hand washing, social distancing, checking how we feel.” -Female nurse 2, outpatient site 2*

### Theme 3: Adaptation process and reaching covidianidad

“Covidianidad” is a term that became a common part of the local vernacular in early 2021 in the DR, which can be loosely translated from Spanish as “everyday COVID” and reflects a change in the situation whereby taking safety measures to prevent COVID infection originally seen as stressors became perceived as manageable and routine as time went by.

#### Subtheme A: Reaching covidianidad and adaptation through learning

Reflecting on how they coped with the different stressors, HCWs perceived they had successfully adjusted to the new “covidianidad.” Educational activities became a cornerstone for HCWs to decrease levels of anxiety and stress. A particular challenge specifically for outpatient participants became discerning trustworthy from unreliable information about COVID, in the context of information overload and misinformation. While inpatient HCWs referred to educational activities organized by their health center as a trustworthy source of information, outpatient HCWs more commonly referred to sources of information like news bulletins and governmental guidance as resources for COVID-19 patient management and safety procedures. However, all agreed that becoming more knowledgeable about COVID-19 allowed them to alleviate their sense of fear and anxiety during the initial stages of the pandemic.

*“Information became clearer, more reliable; we had more strategies, and things normalized, adapting to the pandemic way of life or ‘covidianidad’ as people call it. We learned to live in the pandemic.” -Female psychologist 1, outpatient site 1*

*“All of us learnt something: from celebrating virtual birthdays, which we’ve never done before, to protecting ourselves, our families—we’ve been living ‘covidianidad´.” -Female doctor 1, outpatient site 1*

*“Even if our responsibilities were the same, we’ve had to adjust all we do to fit the new ´covidianidad´.” -Male psychologist 1, outpatient site 2*

#### Subtheme B: Greater use of digital tools in their personal and work lives

The initial stages of the pandemic were characterized by a surge in the use of digital tools. All HCWs expressed using digital communication tools such as WhatsApp to maintain communication with their families, enabling their sense of social support. However, we found instances in which doctors explained that being in digital contact with their families did not substitute for in-person interactions. Other uses of digital tools came in the form of telemedicine and online learning. We found the use of telemedicine mostly discussed by the outpatient sites, and especially in the area of mental health counseling. For online learning, all HCWs expressed using their cellphones or other devices to access educational material and obtain information, despite declaring social media as an unreliable source of information in terms of COVID-19 knowledge.

*“Online course and cellphones were fundamental, because you can access information you can’t physically have contact with.” -Male doctor 1, inpatient site 1*

*“The pandemic taught us to use technology like zoom, videocalls better.” -Female doctor 4, inpatient site 1*

*“We searched for platforms we could use for our services and activities with our patients.” -Female psychologist 1, site 1*

#### Subtheme C: Implementing safety measures

Despite the negative effects of the pandemic, several HCWs highlighted how, in some cases, implementing safety measures provided an unforeseen positive side effect in their lives. Outpatient HCWs mentioned that, during the initial stages of the pandemic, where confinement was mandatory by the authorities, they felt that they became closer to their families, finding ways to spend their days at home together. For inpatient HCWs, however, the most common positive side effect was having more time to care for themselves when they were not at the hospital.

*“We spent more time together, coming up with things to do at home.” -Female CHW 4, outpatient site 1*

*“I’m closer to my children, more than anything, because we got to spend more time together than before the pandemic.” -Female doctor 2, outpatient site 2*

*“Curfew helped. There was no one on the streets drinking alcohol, looking for trouble; we saw less of that.” -Male doctor 1, inpatient site 1*

*“Because of the curfew, I have more time to take care of me than I could before.” -Female doctor 2, inpatient site 2*

#### Subtheme D: Vaccine availability

News of the development of the COVID-19 vaccine was initially met with mixed perceptions from HCWs in our study. Just over half of our participants felt apprehensive and doubtful of receiving the vaccine, mostly due to the array of information and misinformation that surrounded its development. In this respect, outpatient HCWs appeared to be more susceptible to the misinformation and confusion created by person-to-person information and rumors than inpatient HCWs, who mostly described the development of the vaccine as “a ray of hope” that the pandemic may end soon. However, much of this uncertainty dissipated as more information became available, and by the time of the interviews, most HCWs had received at least one dose of the COVID-19 vaccine. Almost half of the participants shared feeling better and safer after getting the vaccine, suggesting that getting the vaccine was perceived as protective by some of our participants.

*“People said if you got the vaccine, you´d die. So, other people became scared.” -Female nurse 1, outpatient site 1*

*“They said the vaccine was the “666 signal,” or it would cause infertility.” -Female doctor 1, outpatient site 2*

*“You heard all these myths: that it was a chip, that the vaccine was killing people.” -Female doctor 4, inpatient site 1*

*“It was a ray of hope. So much has happened, but at least the vaccine can stop this virus from spreading.” -Female doctor 2, inpatient site 1*

*“When I heard about the vaccine, I thought it was a solution.” -Female doctor 4, inpatient site 1*

*“As long as we keep using the vaccine, you feel a bit more comfortable.” -Male psychologist 1, outpatient site 2*

*“With the vaccine, all I know is that I have **some** protection, at least.” -Female doctor 2, inpatient site 2.*

### Theme 4: Future perspectives about COVID and mental health in the Dominican Republic

#### Subtheme A: Challenges to overcome regarding COVID-19 pandemic

We asked HCWs about the most prevalent challenges associated with overcoming the COVID-19 pandemic. For all HCWs, the most prevalent challenge was population behavior regarding safety measures. Population behavior was described as frustrating by all HCWs. There was a strong perception that people not adhering to safety measures were endangering everyone around them. By many HCWs, this reluctance to maintain safety measures was described as a “cultural problem” rather than lack of education or knowledge among the general population.

*“A lot of people don’t protect themselves; one is doing all to protect oneself, and suddenly, someone comes and exposes you unnecessarily.” -Female psychologist 2, outpatient site 1*

*“It’s a cultural thing. Dominican people will do what they want; they think this is all joke.” -Female doctor 2, outpatient site 2*

*“No matter how many times you explain it to them, they use their masks wrong, covering only their mouth. They don’t want the vaccine.” -Female doctor 3, inpatient site 2*

#### Subtheme B: Challenges to overcome regarding HCWs’ mental health

We asked HCWs about mental health-related challenges associated with COVID-19, both for HCWs and the general population. While asked separately, the answers to both questions were the same, centered around the prevalent stigma associated with seeking mental health services, which is perceived as negative and detrimental to the reputation of any individual. In addition to this, participants also highlighted differences in access to mental health services; outpatient HCWs experienced better access to mental health services than inpatient HCWs. Inpatient HCWs expressed that while they knew where to go for mental health services, their health center did not actively encourage or facilitate access to visits with a mental health professional.

*“People still have this idea of ‘why would I go to a psychologist? I’m not crazy’ or [HCWs] usually think ‘I work in the medical area; I can take care of myself. I know what to do,’ and don’t look after their mental health properly.” -Female psychologist 1, outpatient site 1*

*“It’s a bit difficult, the moment you go [to mental health services], people label you as crazy.” -Female doctor 2, inpatient site 2*

*“When you refer patients to mental health, relatives get uncomfortable; ‘why mental health? he’s not crazy’ is what they say.” -Female doctor 2, outpatient site 2*

*“Nobody’s ever told me ‘We’re going to offer therapy for you’ here.” -Female doctor 1, inpatient site 1*

*“We’re not offered psychological consultation here.” -Female nurse 1, inpatient site 2*

When asked about the characteristics that their ideal mental health services would have, HCWs expressed preferences for periodic visits that could be incorporated into their work schedule. These visits could be arranged in coordination between the health center and the Ministry of Public Health to support HCWs’ mental health. This was expressed as being important to support HCWs’ mental health at work, to help ensure that stress at home did not impact their work, and vice versa.

## Discussion

We explored Dominican HCWs’ experiences and perceptions of their mental health during the pandemic, finding issues that were consistent with those seen in other populations as well as some that seem unique to the Dominican setting. Following an initial adjustment period, Dominican HCWs expressed settling into a state of “covidianidad,” a term adopted by the population to refer to refer to adjusting to life in the pandemic.

As COVID-19 cases increased, HCWs in the DR expressed feeling powerless and defenseless due to little knowledge about COVID-19 and fear of the disease, which is consistent with the literature [10, 12]. Uncertainty presented a particular challenge for HCWs, as COVID-19 guidelines often changed swiftly and informal networks circulated misinformation, fomenting mistrust of medical knowledge [14]. Fear of the disease was primarily focused on HCWs’ loved ones with comorbidities that made them vulnerable rather than fear of HCWs becoming infected themselves, much like in other settings [9, 15, 18]. We found an unexpected fear expressed that was not commonly reported in the literature, where HCWs were worried about their children becoming asymptomatic vectors of the disease. Similar to HCWs from Iran [12] and Nepal [17], Dominican HCWs either witnessed or experienced a sense of stigma by the general population, who perceived HCWs as “carriers of COVID”. Specifically, HCWs from ambulatory settings expressed bewilderment that they were met with stigma despite not even working directly in a COVID-19 unit. These findings reflect results from studies proposing that COVID-19 effects were not limited to frontline responders [8, 49, 50]. A notable finding, however, is that although HCWs felt stigmatized, unlike other reports where stigma weighed heavily on the perceived mental health of HCWs [51, 52], our participants were able to rationalize the likely source of this stigmatization, understanding that physically distancing themselves from HCWs was a way for the general population to feel that they were protecting themselves. A possible explanation for the difference between our participants and other findings in the literature may be related to other countries experiencing much more aggressive forms of stigma than the ones expressed by our participants [53–56].

Another significant professional change for HCWs was the way to approach and treat patients. Like previous studies [57–59], HCWs reported that patients reacted with shock, fear and reluctance to accept a positive COVID-19 diagnosis. Dominican culture is adamant in family members having an active role in supporting hospitalized patients and accompanying patients throughout their hospital stay. This custom is in direct contradiction with COVID-19 prevention guidelines, creating a tense and negative atmosphere that affected HCWs and patients alike. Family members and patients adjusting to COVID-19-specific visiting guidelines has been explored in the literature [60], as has the effects of isolation during hospitalization for COVID-19 treatment, a topic widely described as traumatic [57–59, 61]. Much like experiences of Iranian nurses [15], HCWs in the DR perceived the mental state of patients and their relatives as fragile, which in turn became detrimental to the HCWs’ psyche as an added factor to take into consideration while fulfilling their duties, since prior to COVID-19 patient’s relative active role in the care of the patients had a positive effect for both patients and HCWs. Also in concurrence with the literature [9, 21], another barrier to providing patient care was using PPE.

Additional stressors stemmed from the changes in HCWs’ personal lives while implementing safety measures in response to the pandemic. Enacting safety measures at home was perceived as both necessary to stay physically healthy but also cumbersome to sustain over time, causing HCWs to feel challenged by a constantly changing environment. Aside from social distancing and isolation, stressors included loss of income and low salaries. Our results parallel reports on the effects of isolation [6] and the economic impact of COVID-19 [1, 3].

In contrast to stressors, we were also able to identify protective factors that enabled HCWs to continue their work. The most significant protective factor was receiving social support, most commonly from family members. Social support has previously been highlighted as a protective factor associated with mental health outcomes during the COVID-19 pandemic [9, 19, 21]. Our findings also mirror findings from studies across the globe that professional motivation became a protective factor for HCWs [9, 10, 12]. Unlike some studies in the literature [10], our study found no negative connotation to this sense of calling. Also diverging from previous literature from Iran [21] and India [10], and despite DR being a predominantly Catholic country, spirituality and religion were barely mentioned by HCWs during the interviews as a protective factor. Building on these protective factors, HCWs also expressed the importance of having effective management and leaders during times of crisis that can provide quick and appropriate responses and strategies to ease the sense of hopelessness and confusion among HCWs. These findings add to the evidence of how a positive work environment [9, 16, 21, 62] and better understanding of COVID-19 prevention and management [5, 16, 18] were protective factors to HCWs’ mental health, alleviating the initial sense on uncertainty suffered by HCWs.

Mentioned as both a protective factor and one of the cornerstones in the adaptation process was the availability of the vaccine. We found conflicting perceptions regarding vaccine acceptance, with outpatient HCWs more skeptical than inpatient HCWs about the development of the vaccine, mainly due to misinformation and information overload from informal sources. These perceptions have also been reflected in the literature [63–66]. It is notable that from those who were vaccinated at the time of the interview, a little over half felt more protected, inferring a protective effect of vaccination on HCWs’ perceived mental health.

Reaching “covidianidad” seemed to hinge on four cornerstones of the adaptation process: identifying reliable sources of information, leveraging technologic supports, vaccine development, and positive aspects of safety measures. Identifying reliable sources of information and acquiring more knowledge about COVID-19 management allowed HCWs to regain a sense of control lost in the initial stages of the pandemic. Technology played a big role in adapting to the COVID-19 pandemic, both personally and professionally. Personally, technology meant not feeling as isolated and away from loved ones and decreasing effects of social distancing and isolation, whereas professionally, the surge of telemedicine helped ensure services continuity for many healthcare centers, as found in the literature across the globe [67–72]. For our study, however, it was outpatient HCWs who were keener on discussing the use and advantages of telemedicine caring for patients, while inpatient HCWs mainly discussed using technology at work to enhance communication with colleagues. This could be due to a lack of the necessary structure in telemedicine in a country like DR, where last reports indicate that while over 90% of Dominicans have access to the internet and/or mobile phones, only 12% report constant access to broadband on their smartphones, a common barrier among low- and middle-income countries [73–76].

Despite HCWs transitioning into “covidianidad”, our findings identified major perspectives and challenges regarding the COVID-19 response: it is HCWs’ perception that the major challenge to overcome COVID-19 is getting the general population to adhere to safety measures, including getting everyone vaccinated, especially young people [66, 77, 78]. This topic was described as frustrating for all HCWs, as they felt that safety measures and protecting ourselves was everyone’s responsibility, and how a true end to the pandemic will never be achieved unless the general population does their part. We also asked HCWs about the challenges for sustaining the Dominican population and HCWs’ mental health and, surprisingl,y the response to both questions was quite similar. HCWs expressed two major factors: hesitancy towards seeking mental health services, and limited opportunities and facilitators to accessing these services. Stigma towards seeking mental health care is documented all over the globe, and often more pronounced in low- and middle-income countries [79–82]. Recurring barriers to accessing care include social stigma for individuals who need mental health services [83, 84] and not knowing where to seek services [85, 86], a sentiment that was reflected in our findings, where some of our interviewees expressed not having ready access to mental health services. Further research and interventions could focus on increasing access to online mental health services among Dominican HCWs, putting emphasis on inpatient settings. Online mental health services is a tool that has been explored as beneficial and attractive by HCWs in other countries [68, 87, 88].

This study has several strengths. First, we include multiple healthcare sites from two provinces in the Eastern DR. These sites are like many public healthcare settings nationwide, though the extent of transferability of themes is unknown. Another strength is the variety of occupations included in our sample. Previous studies have mainly focused on nurses and doctors. Our study is different as we included CHWs and psychologists, adding their experiences to the existing literature. Additionally, our study looks beyond the professional aspect of being a HCW and explored the consequences of the pandemic on HCWs’ personal lives, further enforcing the double burden of risk factors HCWs have been exposed to during the pandemic.

Limitations include the possibility that the opinions and conclusions drawn from those who were available and interested in participating in an interview are dissimilar from those unavailable and/or uninterested, as transferability can never be assured. Our results are also limited in that the interviews took place from April to August 2021, one year after DR’s first COVID-19 case was diagnosed in March 2020. As such, our qualitative results regarding the start of the pandemic could be influenced by recall bias. Nonetheless, the results for this study provide valuable information: the interviews followed a then/now pattern adequate for describing how HCWs experienced and adapted to the pandemic, allowing participants to reflect on the lasting effects of the COVID-19 pandemic on their mental health.

## Conclusion

Our study investigated the mental health status of Dominican HCWs, an underrepresented population in the literature. Our results add to the growing evidence about the impact of COVID-19 on the lives of healthcare workers. The changes resulting from the COVID-19 pandemic caused HCWs to experience stress, anxiety, and burnout during the early stages of the pandemic, which affected their personal and professional lives. We also identified social support, sense of duty, and institutional organization as protective factors when facing an emerging pandemic. The effects of the COVID-19 pandemic are likely to have a lasting impact on the mental health of Dominican HCWs. Further research should be conducted to inform and develop interventions to reduce stigma associated with seeking mental health services and to facilitate HCWs’ access to services that can improve their mental health.

## Supporting information

S1 Text Interview guide for the participants. (DOCX)

S2 Text Questionnaire of global research inclusivity. (DOCX)
