# Supplementary material for: Reaching “covidianidad”: A qualitative study of the impact of the COVID-19 pandemic on the perceived mental health of health care workers in the Dominican Republic
Source: PLOS Glob Public Health. 2023 Dec 1;3(12):e0002652. doi: 10.1371/journal.pgph.0002652 (PMC10691706; doi:10.1371/journal.pgph.0002652)
Supplement: S1 Text — (DOCX) [file pgph.0002652.s001.docx]

**DR HCW Interview Guide**

*We are interested in understanding your experience as a health care worker during the COVID-19 pandemic. First, we would like to hear about the ways that the pandemic has affected your life in and out of work. Then, we would like to hear your thoughts about what types of supports would be most useful to you and other health care workers during this time. It is important for you to know that there are no right answers; we are most interested in your opinion, as an expert in what you do. I will start by asking you a bit about how COVID-19 has affected your professional life.*

**Work changes**

- Tell me about how your day-to-day work life has changed as a result of COVID-19.
  - *Probe for changes in your job responsibilities (if yes, describe), changes that have been made in how you do your work (if yes, describe)*

**Work environment and COVID-19**

- Tell me about how you feel your institution has responded to the pandemic.
  - *Probe for support from leadership, how things have changed in workplace, sense of safety and protection at work, sense of job security, job satisfaction, burnout, motivation for working*
- How have things changed at work since the start of the pandemic? How are your stress levels about the pandemic compared to how they were in March 2020 when the pandemic started?
- What have been the biggest barriers to feeling safe in terms of COVID-19? What are your biggest concerns with regard to the virus?
  - *Probe for insufficient access to PPE, patients not cooperating and/or not following population safety guidelines, issues with co-workers and staff, public transportation to get to work, family members being exposed, etc.*
- How has your approach to your work changed as a result of this pandemic? How has you’re your approach to work changed?
  - *Probe for feeling more/less pride in work, more/less passionate about job, increase in missed days of work*
- What have been the biggest factors motivating you to continue working as a health care worker?
  - *Probe for pride in work, sense of responsibility, sense of altruism, monetary incentives, enjoyment of the work and/or contentment with work environment/leadership*

**Other topics**

- Some people have described feeling stigmatized as healthcare workers during the COVID-19 pandemic. If you have felt stigma against you during the pandemic due to your role as a healthcare worker or you know of colleagues who have experienced this stigma, please tell me about that.
- From your perspective as a health care worker, what are the greatest challenges facing the DR right now with regard to COVID-19?

*Thank you for sharing these details about your experiences at work. Now, I would like to talk a bit more about how COVID has impacted your life more broadly, including outside of work.*

**Mental health**

- Tell me about your overall wellbeing since the pandemic and how it compares to before the start of the pandemic?
  - *Probe: changes in mental health? (mood, anxiety, enjoyment in things, ability to relax, sleep)*

**Life changes**

- Apart from work, what other changes in your life have occurred as a result of COVID? Tell me about any logistical difficulties that you have encountered during your daily life since the beginning of COVID.
  - *Probe for changes in home life, child care, transport, taking care of parents*

**Social support**

- Tell me about your social support since the start of the pandemic. In what ways have you been more socially isolated or more connected? Where is your strongest source of social support coming from? (colleagues, spouse, family, parents, children, friends, etc.)

**Biggest challenges**

- What would you say was your biggest concern at the beginning of the pandemic, and what is your biggest concern now?
- Thinking about challenges inside and outside of work, what are your biggest stressors right now? Are your biggest life stressors related to COVID or related to other things?
  - *Probe for financial difficulties, concerns about job, childcare complications, having to work overtime, PPE issues and fear about COVID stuff, stress from other jobs, loss of friends or family members, etc.*

**Coping**

- Tell me about how you feel that you have been handling/juggling all of the stressors in your life. What have you been doing to cope? What coping strategies have you tried that have helped, and what have you tried that hasn’t helped?
  - *Probe for exercise, reading, praying, walking, talking to friends, sharing jokes, talking to a therapist, watching TV, practicing a hobby, playing games*
- Tell me about any changes that you have noticed in the way you take care of yourself since the start of the pandemic, either for better or for worse.
  - *Probe for diet, exercise, drinking, smoking, self-care*

*Thank you for sharing your thoughts on some of the ways that the COVID-19 pandemic has affected you and for talking about some of the biggest challenges in recent months. We would now like to talk about ways of better supporting you. Specifically, we would like to you’re your thoughts about how your workplace could better support you and about possible strategies to support the mental health of you and other health care workers during this time.*

**Mental health support**

- What could your organization do to support your mental wellbeing during this time? What changes or supports could be put in place to improve your work life and/or work performance?
  - *Probe for more employee recognition, more PPE, establishing safety protocols for employees, access to psychological services/support, increased access to equipment/supplies (pulse oxygen machines, medication, ventilators), more education about the signs and symptoms of anxiety and trauma, more education about COVID-19, clearer details about what to do when feeling sick and who to speak with when feeling burdened by emotions or stress, financial incentives or bonuses, etc.*
- Tell me about access to mental health counseling at your organization, including what services are available, the usefulness of these services, and the extent to which they are utilized
  - *If there is a service, probe for how often staff use the service, why it is/is not used (i.e. biggest barriers to using it, what gets in the way)*
  - *If there is not a service, probe for whether or not staff would use a service like this if it was available, what might be the biggest barriers to utilizing it (i.e., what might get in the way*
- What broader supports (outside of your workplace) would be helpful in supporting you during this time? This could be support related to mental health, or related to other stressors in your life
  - *Probe for supports related to financial stressors, stressors related to your living situation, children, etc.*
- What do you think the biggest challenges are in terms of mental health of health care workers in the Dominican Republic?
- Do you have any additional comments about the topic of mental health within the Dominican Republic?

**Closing remarks**

- Is there anything else that you think would be relevant to our conversation today that we have not mentioned?
